# Predefined-Time Sliding Mode Control of Robotic Manipulators via Artificial Delay Feedback and Reinforcement Learning

**DOI:** 10.3390/s26113543

**Published:** 2026-06-03

**Authors:** Lei Zhang, Jianli Wang, Jialong Wang, Jintong Lu, Peng Li

**Affiliations:** 1 Institute of Complexity Science, Henan University of Technology, Zhengzhou 450001, China; zhanglei@haut.edu.cn (L.Z.); jianli@stu.haut.edu.cn (J.W.); 2 College of Electrical Engineering, Henan University of Technology, Zhengzhou 450001, China; wangjialong@stu.haut.edu.cn (J.W.); lujintong@stu.haut.edu.cn (J.L.)

**Keywords:** robotic manipulators, predefined-time control, nonlinear sliding mode control, trajectory tracking control, reinforcement learning, sensor-based control, joint position sensors

## Abstract

To address the rigid temporal constraints and high-precision trajectory tracking requirements in modern industrial automation (e.g., high-speed pick-and-place or collaborative assembly), this paper proposes a novel composite control strategy for robotic manipulators that integrates Actor–Critic reinforcement learning with predefined-time sliding mode control (PTC-RLC). Existing predefined-time control (PTC) schemes usually rely on excessively large switching gains when dealing with strong disturbances, which easily triggers severe chattering in the system’s actuators and degrades dynamic performance. To this end, a novel predefined-time sliding surface based on artificial delay feedback is designed, ensuring that the position tracking error can strictly converge within a user-explicitly set time $T_{c}$ regardless of the system’s initial states, thereby significantly enhancing temporal determinism. Meanwhile, a reinforcement learning agent based on the Actor–Critic architecture is constructed to approximate and dynamically compensate for the system’s lumped unknown dynamics and external disturbances online, minimizing the control law’s reliance on large robust gains. Based on Lyapunov stability theory, the semi-global uniform ultimate boundedness of the closed-loop system is strictly proved. Numerical simulation results demonstrate that under severe operating conditions with parameter mismatches and time-varying disturbances, the proposed control strategy not only achieves high-precision and singularity-free trajectory tracking within the predefined time, but also effectively suppresses high-frequency chattering phenomena compared to the traditional non-singular terminal sliding mode control (NTSMC), outputting a smoother control torque and demonstrating strong potential for practical engineering implementations.

## 1. Introduction

Robotic manipulator systems are characterized by strong nonlinearity, high coupling, and sensitivity to external disturbances; therefore, achieving high-precision trajectory tracking remains a critical problem in nonlinear control. In early studies, Lin et al. [1] designed a robust controller for robotic manipulators based on optimal control theory; Ahmad et al. [2] proposed a proportional–integral sliding mode control method, which improved the steady-state performance of the system by introducing an integral term. In addition, some studies have attempted to use adaptive control strategies to cope with model uncertainties, but performance degradation still exists in complex disturbance environments.

Fundamentally, the practical implementation of high-precision trajectory tracking is inextricably linked to the performance of the underlying sensor-based feedback control systems. Real-time state variables, such as joint angular positions and velocities, must be continuously acquired via high-precision joint sensors (e.g., optical rotary encoders). However, in actual engineering deployments, physical sensors inevitably introduce measurement noise, and the overall robotic sensory system is highly susceptible to external environmental disturbances [3]. Recently, integrating adaptive estimation and learning-assisted architectures with sensor feedback has become a vital trend to improve the motion control of autonomous systems [4]. Therefore, when designing a control strategy for robotic manipulators, it is crucial to ensure the algorithm possesses strong robustness against the inherent uncertainties and dynamic interference present in real-world sensor data acquisition.

To enhance the system’s robustness against uncertainties and external disturbances, sliding mode control (SMC) is widely used in robotic manipulator systems. Man et al. [5] proposed a multi-input multi-output terminal sliding mode control method to achieve asymptotic stability of the system state; Levant [6] theoretically developed high-order sliding mode control methods; and Utkin [7] systematically summarized the application of sliding mode control in engineering. However, traditional sliding mode control is prone to severe high-frequency chattering problems due to the use of discontinuous switching terms. To address this problem, Bartolini et al. [8] weakened the chattering phenomenon by improving the reaching law, and Shtessel et al. [9] systematically analyzed the chattering mechanism and suppression methods from an engineering perspective. To improve the convergence speed, non-singular terminal sliding mode control was proposed. Rathaur [10] designed a singularity-free fast terminal sliding mode controller; Sun et al. [11] introduced neural networks into finite-time sliding mode control to achieve faster convergence performance; and Gao et al. [12] utilized RBF neural networks to approximate system uncertainties online. However, these methods usually rely on large switching gains to ensure finite-time convergence, resulting in prominent chattering problems.

To overcome the problem that convergence time depends on initial states, Zhang et al. [13] proposed a fixed-time sliding mode control method; Polyakov [14] systematically studied fixed-time stability theoretically; and Zuo [15] proposed a singularity-free fixed-time control method. Building upon this, Cao et al. [16] introduced reinforcement learning to achieve fixed-time trajectory tracking control under input constraints. However, fixed-time control methods generally suffer from complex parameter design and conservative upper bounds of convergence time. To achieve explicit adjustment of convergence time, predefined-time control has gradually become a research hotspot. Jia et al. [17] proposed a predefined-time non-singular sliding mode control method; Muñoz-Vázquez et al. [18] studied the predefined-time robust stabilization of robotic systems. Regarding applications in different fields, Yu et al. [19] designed a predefined-time non-singular fast terminal sliding mode for trajectory tracking of underwater vehicles (ROVs); Chen et al. [20] and Zheng et al. [21] achieved predefined-time stabilization control of spacecraft attitude based on observer theory and fully actuated system approaches, respectively. In the field of robotic manipulators and complex robots, Liu et al. [22] proposed a general predefined-time terminal sliding mode control scheme for dual-arm space robots; Xu et al. [23] further explored event-triggered adaptive sliding mode control with predefined-time convergence characteristics; Li et al. [24] applied PTC to conventional robotic manipulator trajectory tracking; Wang et al. [25] provided a systematic review of this field; and Shao et al. [26] studied the predefined-time stability of uncertain systems. Furthermore, Song et al. [27] systematically solved the prescribed-time stabilization problem of strict-feedback nonlinear systems theoretically, laying a foundation for the generalization of such methods. These studies show that PTC has significant advantages in improving system dynamic performance. However, under complex disturbances, the aforementioned methods usually rely on large robust gains, thereby aggravating the chattering of control inputs.

In industrial automation contexts, such as high-speed pick-and-place operations, automated sorting, and collaborative precision assembly, robotic manipulators are required to complete specified motions within rigid, safety-critical time windows. Under these operating conditions, conventional asymptotic or finite-time control methods become inadequate because their convergence time bounds heavily depend on or grow with the initial states of the system, which are often uncertain or variable on a real-world production line. Predefined-time stability overcomes this limitation by decoupling the settling time from the initial conditions, allowing control engineers to explicitly pre-program the exact convergence time $T_{c}$ directly into the software architecture, thereby guaranteeing predictable cycle times and superior dynamic performance. It is worth noting that although the integration of sliding mode control with reinforcement learning has been previously explored in the literature [28,29,30], those schemes typically still rely on high-gain switching terms even after neural network compensation, and rarely guarantee a strictly user-prescribed convergence time. The strategy proposed in this paper differs in that it concurrently achieves (i) strict predefined-time convergence via a smooth artificial-delay sliding surface, (ii) online Actor–Critic compensation that reduces dependence on large robust gains, and (iii) a singularity-free, low-chattering control input suitable for industrial-grade actuators.

In recent years, with the development of data-driven control, reinforcement learning (RL) has gradually been introduced into nonlinear system control. Vamvoudakis and Lewis [31] proposed an online Actor–Critic control framework; Lewis et al. [32] systematically summarized its application in control; and Zhao et al. [33] provided a review of reinforcement learning-based control methods. Modares et al. [34] proposed a framework using RL to solve the optimal control of nonlinear systems with partially unknown dynamics. In the field of robotic manipulator control, Liu et al. [35] achieved trajectory tracking using reinforcement learning; Liu et al. [28] specifically designed a predefined-time tracking controller based on the Actor–Critic algorithm for n-DOF manipulators; and Wang et al. [29] introduced the Actor–Critic structure into the predefined-time control of nonlinear systems. To address modeling uncertainties, Hao et al. [36] significantly improved system robustness using RBF neural network adaptive compensation, while Wang et al. [37] combined fuzzy neural networks with the Actor–Critic architecture to enhance the system’s adaptive capability against external disturbances. In addition, Sun et al. [30] combined reinforcement learning with sliding mode control, reducing chattering to a certain extent. However, existing research mostly utilizes reinforcement learning as an auxiliary compensation means and still relies on high-gain sliding mode terms to guarantee robustness, thus failing to fundamentally resolve the chattering problem.

In summary, existing methods have not yet simultaneously achieved the following three objectives: predefined-time convergence, low-chattering control input, and low reliance on high robust gains. Therefore, how to achieve a control strategy with low chattering and good engineering feasibility while ensuring strict predefined-time convergence remains an unresolved problem.

To address the above problems, this paper proposes a predefined-time sliding mode control method integrating reinforcement learning (PTC-RLC). This method achieves strict error convergence within a user-defined time by constructing a predefined-time sliding surface based on smooth artificial delay feedback; meanwhile, it introduces an Actor–Critic network to approximate lumped system uncertainties online, effectively compensating for the physical sensor measurement uncertainties and dynamic disturbances, thereby reducing the controller’s reliance on large robust gains and weakening chattering from the mechanism. Based on Lyapunov theory, the semi-global uniform ultimate boundedness of the closed-loop system is proven.

The remainder of this paper is organized as follows: Section 2 introduces the system model. Section 3 presents the preliminaries and controller design. Section 4 performs the stability analysis. Section 5 verifies the effectiveness of the proposed method through simulation. Section 6 provides the conclusion.

## 2. Model Description

**Figure 1:**
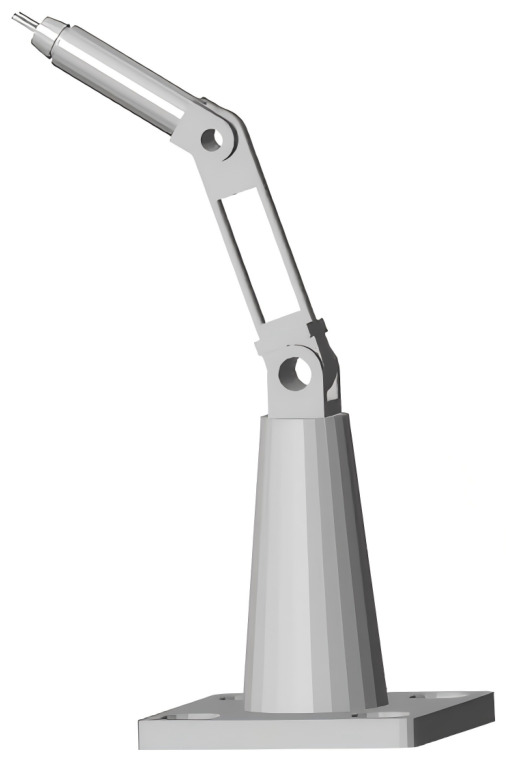
Schematic diagram of the 2-DOF robotic manipulator model.

While the proposed control architecture is universally applicable to *n*-DOF manipulators, where the per-joint sliding-surface computation and the Actor compensator scale linearly with the number of joints, a 2-DOF robotic manipulator model is selected in this section for explicit mathematical illustration and subsequent simulation verification. Consider a 2-DOF robotic manipulator model, whose structure is shown in Figure 1.

The Lagrangian dynamic equation of an *n*-degree-of-freedom (DOF) robotic manipulator affected by parameter uncertainties and external disturbances is formulated as follows:

(1) $$M\left(q\right)\ddot{q}+C\left(q,\dot{q}\right)\dot{q}+G\left(q\right)=\tau \left(t\right)+d\left(t\right)$$

where $q\left(t\right),\;\dot{q}\left(t\right),\;\ddot{q}\left(t\right)\in R^{n}$ are the joint angular position, velocity, and acceleration vectors, respectively; $\tau \left(t\right)\in R^{n}$ is the control torque; $d\left(t\right)\in R^{n}$ denotes the external lumped generalized disturbances, encompassing joint friction, payload variations, and unmodeled external forces; and M(q), $C(q,\dot{q})$, and G(q) represent the inertia matrix, Coriolis/centrifugal matrix, and gravity vector, respectively. By decomposing these system matrices into nominal parts (subscript 0) and uncertain parts (subscript Δ), Equation (1) can be compactly rearranged as

(2) $$\ddot{q}=M_{0}^{-1}\tau +l\left(t\right)$$

where $M_{0}\left(q\right)$ is the nominal invertible inertia matrix, and $l\left(t\right)=\left(M^{-1}-M_{0}^{-1}\right)\tau +M{\left(q\right)}^{-1}\left[-C\left(q,\dot{q}\right)\dot{q}-G\left(q\right)\right]+M{\left(q\right)}^{-1}d\left(t\right)$ represents the lumped system uncertainty, which encapsulates unmodeled dynamics, parameter variations, and external disturbances. To facilitate the subsequent predefined-time controller design, the trajectory tracking error vectors are defined as follows. Let $q_{d}\in R^{n}$ represent the ideal continuous reference trajectory. The position tracking error $e_{1}\in R^{n}$ and velocity tracking error $e_{2}\in R^{n}$ are defined as

(3) $$\left\{\begin{array}{c} e_{1}=q_{d}-q\\ e_{2}={\dot{q}}_{d}-\dot{q} \end{array}\right.$$

Taking the time derivative of the error vectors yields the foundational error evolution dynamics:

(4) $$\left\{\begin{array}{c} {\dot{e}}_{1}=e_{2}\\ {\dot{e}}_{2}={\ddot{q}}_{d}-\ddot{q} \end{array}\right.$$

By substituting the isolated system dynamics from Equation (2) into the acceleration error, the complete closed-loop error evolution equation can be explicitly derived as

(5) $${\dot{e}}_{2}={\ddot{q}}_{d}-M_{0}^{-1}\tau -l\left(t\right)$$

where $e_{2},\;{\ddot{q}}_{d},\;\tau ,$ and $l\left(t\right)\in R^{n}$ are *n*-dimensional vectors. Assume that the reference trajectory $q_{d}$ and its second derivative ${\ddot{q}}_{d}$ are continuous and bounded. To facilitate the subsequent controller design, the necessary mathematical foundation for function approximation and specific control objectives will be introduced in Section 3.

## 3. Preliminaries and Controller Design

In this section, Radial Basis Function Neural Networks (RBFNNs) are employed to approximate the unknown lumped nonlinear uncertainty l(t) by virtue of their universal approximation property [12]. Benefiting from this capability, any continuous function can be modeled by bounded neural network weights and approximation errors. In the subsequent composite controller design, the Critic network and the Actor network will directly utilize specific Gaussian basis function vectors, denoted as $\varphi_{c}\left(e_{1}\right)$ and $\psi_{a}\left(z\right)$, respectively, to balance structural simplicity and computational efficiency.

**Lemma 1** 
(Robust Fixed-Time Reaching Law Based on Nonlinear Saturation Term [38]). *Consider the following perturbed scalar system:*

(6) $$\dot{x}=-k{\left\lfloor x\right\rceil }^{\frac{\lambda x^{2}}{1+\mu x^{2}}}+d\left(t\right)$$

*where the symbol is defined as ${\left\lfloor x\right\rceil }^{p}={\left|x\right|}^{p}\mathrm{sign}\left(x\right)$, with x denoting a generic scalar state variable (distinct from the robotic joint vector q in Section 2). The disturbance satisfies the bounded condition |d(t)|≤δ. If the control parameters satisfy $\chi =\frac{\lambda }{1+\mu }>1$, and the gain k is sufficiently large such that $k>\delta e^{\frac{\lambda }{2e}}$, then the system is fixed-time stable, and its convergence time is strictly controlled by parameters independent of the initial states. In computer simulations, to attenuate high-frequency chattering, a continuous saturation function sat(x/ϵ) can be used instead of the sign function.*

**Lemma 2** 
(Predefined-Time Convergence Based on Smooth Artificial Delay Feedback). *Inspired by the delayed feedback concept in [39] and based on the framework in [40], consider the following scalar system with an artificial delay:*

(7) $$\dot{\zeta }\left(t\right)=a\zeta \left(t\right)-K\left(t\right)\zeta \left(t-h\right)$$

*where a∈R, and the delay h>0. The time-varying feedback gain K(t) is designed as*

(8) $$K\left(t\right)=R_{h}\left(t\right)e^{ah}We^{-2at}$$

*where $R_{h}\left(t\right)$ is a globally smooth Bump function defined as*

(9) $$R_{h}\left(t\right)=\left\{\begin{array}{cc} \exp\left(-\frac{M}{\left(t-h)(2h-t\right)}\right), & t\in (h,2h)\\ 0, & t\notin (h,2h) \end{array}\right.$$

*The constant M>1 is used to ensure boundary smoothness and prevent numerical overflow. The normalized integral weight W is defined as*

(10) $$W={\left(\int_{h}^{2h}R_{h}\left(\theta \right)e^{-2a\theta }d\theta \right)}^{-1}$$

*Then, the above system is prescribed-time stable, and the system states will strictly converge to zero at the predefined time t=2h and thereafter; i.e., ζ(t)≡0,∀t≥2h.*

The control objective of this paper is as follows: for a 2-DOF robotic manipulator system subject to lumped uncertainties l(t), combine the aforementioned predefined-time theory and reinforcement learning (Actor–Critic) technique to design a composite control law τ(t), such that the joint position *q* can achieve singularity-free, high-precision tracking of the desired trajectory $q_{d}$ within a user-predefined exact time $T_{c}$.

### 3.1. Critic Network Design

To achieve optimal tracking performance, a reinforcement learning framework is introduced in this paper. First, an infinite-horizon discounted cost function J(t) is defined to quantify the control performance:

(11) $$J\left(t\right)=\int_{t}^{\infty }e^{-\gamma_{d}\left(\tau -t\right)}r\left(\tau \right)d\tau$$

where J(t) represents the infinite-horizon discounted cost function that quantifies the long-term control performance of the reinforcement learning agent, and $\gamma_{d}>0$ denotes the constant discount factor (renamed from the conventional χ to avoid notational conflict with the stability parameter χ in Lemma 1). The instantaneous utility function is designed as $r\left(t\right)=e_{1}^{T}Qe_{1}+\tau^{T}R\tau$, where *Q* and R are positive semi-definite and positive definite weight matrices, respectively. It is worth noting that the utility function explicitly incorporates the joint position tracking error $e_{1}$ defined in Equation (4). By minimizing the long-term cost function J(t), the reinforcement learning framework successfully minimizes the trajectory tracking error while penalizing excessive control energy consumption, thereby achieving a balance between tracking accuracy and actuator efficiency.

Since the analytical form of J(t) is usually unobtainable, a Critic neural network is employed for online approximation. The estimated value function is denoted as $\hat{J}\left(t\right)=w_{c}^{T}\varphi_{c}\left(e_{1}\right)$, where $w_{c}\in R^{l}$ represents the weight vector, and $\varphi_{c}\left(e_{1}\right)$ is the radial basis function vector. By differentiating it with respect to time, the Bellman residual error ϵ can be derived as

(12) $$\epsilon =r\left(t\right)-\frac{1}{\gamma_{d}}w_{c}^{T}\varphi_{c}+w_{c}^{T}\nabla \varphi_{c}{\dot{e}}_{1}$$

To minimize the objective function $E_{c}=\frac{1}{2}\epsilon^{2}$, the weight update law is designed using gradient descent as follows:

(13) $${\dot{w}}_{c}=-\eta_{c}\frac{\partial E_{c}}{\partial w_{c}}=-\eta_{c}\epsilon \Omega$$

where $\eta_{c}>0$ is the learning rate, and $\Omega =\nabla \varphi_{c}{\dot{e}}_{1}-\frac{1}{\gamma_{d}}\varphi_{c}$ represents the regressor vector. To guarantee the boundedness of weights (i.e., $∥w_{c}∥\leq {\bar{w}}_{c}$), by introducing a parameter projection operator, the update law is reconstructed as

(14) $${\dot{w}}_{c}=\left\{\begin{array}{cc} -\eta_{c}\epsilon \Omega , & \mathrm{if}\;∥w_{c}∥<{\bar{w}}_{c}\;\mathrm{or}\;(∥w_{c}∥={\bar{w}}_{c}\;\mathrm{and}\;\epsilon w_{c}^{T}\Omega \geq 0)\\ -\eta_{c}\epsilon \Omega +\eta_{c}\varrho_{c}, & \mathrm{if}\;∥w_{c}∥={\bar{w}}_{c}\;\mathrm{and}\;\epsilon w_{c}^{T}\Omega <0 \end{array}\right.$$

Through the projection update law, the Critic network weights $w_{c}$ are guaranteed to remain within a predefined compact set, thereby ensuring the boundedness of the estimated value function $\hat{J}\left(t\right)$ and the Bellman error ϵ. Here, $\varrho_{c}$ is the projection correction term, defined as

(15) $$\varrho_{c}=\frac{\epsilon w_{c}^{T}\Omega }{∥w_{c}{∥}^{2}}w_{c}$$

### 3.2. Actor Network and Controller Design

For the trajectory tracking task of the robotic manipulator, the position tracking error for each joint is defined as $e_{1,i}=q_{d,i}-q_{i}$, and the velocity tracking error as $e_{2,i}={\dot{q}}_{d,i}-{\dot{q}}_{i}$. To achieve strict convergence of system errors within a user-defined total time $T_{c}$, this paper defines a piecewise delay parameter as $h=T_{c}/3$. This temporal partitioning scheme splits the entire closed-loop error evolution into three sequential phases of equal duration *h*: the sliding mode reaching phase (t∈[0,h]), the un-delayed exponential decay sliding phase (t∈[h,2h]), and the artificial delay-driven forced convergence phase (t∈[2h,3h]). The choice $h=T_{c}/3$ is dictated by the structural requirement of the delayed-feedback sliding surface. According to Lemma 2, the smooth Bump kernel $R_{h}\left(t\right)$ has compact support on the interval (h,2h); by construction $K_{0}\left(t\right)=K\left(t-h\right)$ shifts this active window to (2h,3h), which serves as the forced convergence interval. Two additional intervals of equal length *h* are therefore required: the first interval [0,h] is reserved for the sliding-mode reaching phase during which the robust term drives $s_{i}\to 0$ in finite time $T_{s}\leq h$ (as ensured by Lemma 1), and the intermediate interval [h,2h] accommodates the un-delayed exponential decay during which $K_{0}\left(t\right)=0$. This three-stage equal-duration allocation simultaneously guarantees the mathematical tractability of the resulting delay differential equation, the singularity-free transition across phases, and strict convergence at $t=3h=T_{c}$.

(16) $$s_{i}\left(t\right)=e_{2,i}\left(t\right)-ae_{1,i}\left(t\right)+K_{0}\left(t\right)e_{1,i}\left(t-h\right)$$

where the time-varying gain coefficient is defined by employing the lag-time window control as $K_{0}\left(t\right)=K\left(t-h\right)$ to ensure that the forced convergence mechanism is activated during t∈[h,3h]. Taking the time derivative of the above sliding variable yields the closed-loop error dynamic system equation:

(17) $${\dot{s}}_{i}\left(t\right)={\dot{e}}_{2,i}\left(t\right)-a{\dot{e}}_{1,i}\left(t\right)+K_{0}\left(t\right){\dot{e}}_{1,i}\left(t-h\right)+{\dot{K}}_{0}\left(t\right)e_{1,i}\left(t-h\right)$$

Substituting the manipulator nominal dynamics ${\dot{e}}_{2}={\ddot{q}}_{d}-v-M_{0}^{-1}l\left(t\right)$ into the above equation (where *v* is the nominal control input to be designed), according to the equivalent control principle of sliding mode control and the nonlinear robust reaching law of Lemma 1, the nominal control input $v_{i}$ is designed as the sum of two parts:

(18) $$v_{i}\left(t\right)=u_{eq,i}\left(t\right)+u_{rob,i}\left(t\right)$$

The equivalent control law $u_{eq,i}\left(t\right)$, aimed at guiding the error along the sliding surface, is designed as

(19) $$u_{eq,i}\left(t\right)={\ddot{q}}_{d,i}-ae_{2,i}\left(t\right)+K_{0}\left(t\right){\dot{e}}_{1,i}\left(t-h\right)+{\dot{K}}_{0}\left(t\right)e_{1,i}\left(t-h\right)$$

The predefined-time robust reaching law $u_{rob,i}\left(t\right)$, which drives the sliding variable to the sliding surface in finite time, is designed as

(20) $$u_{rob,i}\left(t\right)=k_{i}{\left|s_{i}\right|}^{\frac{\lambda s_{i}^{2}}{1+\mu s_{i}^{2}}}\mathrm{sat}\left(s_{i}/\epsilon \right)$$

Since the lumped uncertainty l(t) contains complex unmodeled dynamics and external disturbances, its precise bounds are often difficult to obtain directly. Therefore, this paper introduces the output $f_{NN}$ of the reinforcement learning Actor network for online approximation and dynamic compensation. Combining the inverse dynamics equation of the robotic manipulator, the final composite predefined-time reinforcement learning control law (PTC-RLC) τ(t) is designed as

(21) $$\tau \left(t\right)=M_{0}\left(q\right)\left({\ddot{q}}_{d}-v\left(t\right)-f_{NN}\right)+C_{0}\left(q,\dot{q}\right)\dot{q}+G_{0}\left(q\right)$$

Through the above control law, benefiting from the precise online approximation of the Actor–Critic network and the infinite differentiability ($C^{\infty }$) of the Bump function $R_{h}\left(t\right)$ at the boundaries, the proposed scheme not only fundamentally avoids the singularity dilemma caused by fractional-order powers in traditional fixed-time sliding surfaces, but also enables the system to complete high-precision trajectory tracking within the strict user-predefined time $T_{c}$ when facing sudden load changes or continuous external disturbances.

## 4. Stability Analysis

### 4.1. Error Dynamics and Reconstruction

For convenience of analysis, we denote $e_{1,i}$ and $e_{2,i}$ as the *i*-th components of the error vectors $e_{1}$ and $e_{2}$ respectively.

From the robotic manipulator dynamics, the total approximation error of the Actor network for the lumped uncertainties is defined as $f_{NN,i}$, which consists of the weight estimation error and the inherent reconstruction error $\epsilon_{i}$:

(22) $$f_{NN,i}=W_{a,i}^{T}\psi_{a}\left(z\right)-W_{a,i}^{*T}\psi_{a}\left(z\right)+\epsilon_{i}={\tilde{W}}_{a,i}^{T}\psi_{a}\left(z\right)+\epsilon_{i}$$

Based on the universal approximation property of neural networks, assume the reconstruction error is bounded and satisfies $|\epsilon_{i}|\leq {\bar{\epsilon }}_{i}$. Furthermore, since Gaussian basis functions satisfy $∥\psi_{a}\left(z\right)∥\leq 1$, and the estimated weights are constrained by the parameter projection algorithm to satisfy $∥W_{a,i}∥\leq {\bar{W}}_{a,i}$, the total approximation error is strictly bounded:

(23) $$|f_{NN,i}|\leq ∥{\tilde{W}}_{a,i}∥∥\psi_{a}\left(z\right)∥+|\epsilon_{i}|\leq (∥W_{a,i}∥+∥W_{a,i}^{*}∥)+{\bar{\epsilon }}_{i}:=\Delta_{NN,i}$$

where $\Delta_{NN,i}$ is the physical upper bound of the system residual approximation error. Combining the equivalent control law $u_{eq,i}$ and robust control law $u_{rob,i}$, the dynamic evolution equation of the sliding variable can be expressed as

(24) $${\dot{s}}_{i}\left(t\right)=f_{NN,i}-u_{rob,i}\left(t\right)={\tilde{W}}_{a,i}^{T}\psi_{a}\left(z\right)+\epsilon_{i}-u_{rob,i}\left(t\right)$$

### 4.2. Closed-Loop System Stability Proof

**Theorem 1.** 
*Consider a 2-DOF robotic manipulator system affected by lumped disturbances. Utilizing the predefined-time sliding surface $s_{i}\left(t\right)$ and the composite control law τ(t), the weight update of the reinforcement learning network is given by an adaptive law with a projection operator. If the robust gain is selected to satisfy $k_{rob}>\Delta_{NN,i}e^{\frac{\lambda }{2e}}$, then the closed-loop system satisfies semi-global uniform ultimate boundedness, and the position tracking error $e_{1,i}$ will precisely converge to zero within the user-predefined time $T_{c}=3h$.*

To clearly illustrate the convergence mechanism of the multi-coupled dynamics, this proof divides the system evolution into four sequential phases: network weight boundedness analysis, sliding mode reaching phase, exponential decay sliding phase, and predefined-time forced convergence phase.

Select a comprehensive candidate Lyapunov function of the following form:

(25) $$V\left(t\right)=\frac{1}{2}s^{T}s+\frac{1}{2\eta_{c}}{\tilde{W}}_{c}^{T}{\tilde{W}}_{c}+\sum_{i=1}^{2}\frac{1}{2\eta_{a,i}}{\tilde{W}}_{a,i}^{T}{\tilde{W}}_{a,i}$$

Let $V_{s}=\frac{1}{2}s^{T}s$, and $V_{NN}=\frac{1}{2\eta_{c}}{\tilde{W}}_{c}^{T}{\tilde{W}}_{c}+\sum_{i=1}^{2}\frac{1}{2\eta_{a,i}}{\tilde{W}}_{a,i}^{T}{\tilde{W}}_{a,i}$. Taking the time derivative of V(t) along the system trajectories yields $\dot{V}\left(t\right)=s^{T}\dot{s}+{\dot{V}}_{NN}$.

#### 4.2.1. Phase 1: Boundedness Analysis of Neural Network Weights via Case Classification

To verify the system’s convergence, we separately analyze the derivative of the neural network energy term ${\dot{V}}_{NN}$. Considering the activation conditions of the parameter projection algorithm at the boundaries, the analysis of ${\dot{V}}_{NN}$ must be strictly divided into the following four boundary cases:

*Case 1:* Both the Critic network and Actor network have not triggered the compact set boundary, or triggered the boundary but the gradient direction points inward (i.e., $∥W_{c}∥<{\bar{W}}_{c}$ or $∥W_{c}∥={\bar{W}}_{c}\;\mathrm{and}\;\epsilon W_{c}^{T}\Omega \geq 0$, similarly for Actor). In this case, the projection correction terms $\rho_{c}$ and $\rho_{a,i}$ are inactive:

(26) $${\dot{V}}_{NN}={\tilde{W}}_{c}^{T}\left(\epsilon \Omega \right)+\sum_{i=1}^{2}{\tilde{W}}_{a,i}^{T}\rho_{a,i}\leq (1+∥W_{c}∥∥\Omega ∥)(∥W_{c}^{*}∥+∥W_{c}∥)∥\Omega ∥+2(∥W_{a,i}^{*}∥+∥W_{a,i}∥):=\sigma_{1}$$

*Case 2:* The Critic network is within the boundary, while the Actor network triggers the boundary with its update direction pointing outward. Here, only the Actor network activates the projection correction term:

(27) $${\dot{V}}_{NN}={\tilde{W}}_{c}^{T}\left(\epsilon \Omega \right)+\sum_{i=1}^{2}{\tilde{W}}_{a,i}^{T}\left(\rho_{a,i}-\varrho_{a,i}\right)\leq \left(1+∥W_{c}∥∥\Omega ∥\right)(∥W_{c}^{*}∥+∥W_{c}∥)∥\Omega ∥:=\sigma_{2}$$

*Case 3:* The Critic network triggers boundary correction, while the Actor network is within the boundary. Similarly, we obtain

(28) $${\dot{V}}_{NN}={\tilde{W}}_{c}^{T}\left(\epsilon \Omega -\varrho_{c}\right)+\sum_{i=1}^{2}{\tilde{W}}_{a,i}^{T}\rho_{a,i}\leq 2(∥W_{a,i}^{*}∥+∥W_{a,i}∥):=\sigma_{3}$$

*Case 4:* Both networks are on the boundary with update directions pointing outward. Here, both activate correction terms to cancel the divergent gradients:

(29) $${\dot{V}}_{NN}={\tilde{W}}_{c}^{T}\left(\epsilon \Omega -\varrho_{c}\right)+\sum_{i=1}^{2}{\tilde{W}}_{a,i}^{T}\left(\rho_{a,i}-\varrho_{a,i}\right)=0$$

Summarizing the four cases, the derivative of the neural network energy term is objectively bounded by an upper limit. That is, there exists a positive constant $\Theta =\max(\sigma_{1},\sigma_{2},\sigma_{3},0)$ such that ${\dot{V}}_{NN}\leq \Theta$ always holds. This strictly proves the global boundedness of the Actor–Critic network weights during the adaptive learning process.

#### 4.2.2. Phase 2: Sliding Mode Reaching Phase (t∈[0,h])

Expanding the derivative of the sliding variable part, the overall dissipation inequality of the system is

(30) $$\dot{V}\left(t\right)\leq \sum_{i=1}^{2}|s_{i}|\Delta_{NN,i}-\sum_{i=1}^{2}k_{rob}\left|s_{i}\right|\frac{\lambda s_{i}^{2}}{1+\mu s_{i}^{2}}+1+\Theta$$

Select a sufficiently large robust switching gain such that the dominant term $k_{rob}|s_{i}|\frac{\lambda s_{i}^{2}}{1+\mu s_{i}^{2}}+1\gg \left|s_{i}\right|\Delta_{NN,i}+\Theta$. According to robust control theory, $\dot{V}\left(t\right)<0$ always holds outside the sliding surface. There exists a time constant $T_{s}\leq h$, such that the sliding variable is forcibly suppressed and maintained at $s_{i}\left(t\right)\equiv 0$ within this time.

#### 4.2.3. Phase 3: Exponential Decay Sliding Phase (t∈[h,2h])

After entering the sliding mode (i.e., $\forall t\geq h,s_{i}\left(t\right)=0$), the error dynamics obey the sliding-surface equation:

(31) $${\dot{e}}_{1,i}\left(t\right)=ae_{1,i}\left(t\right)-K_{0}\left(t\right)e_{1,i}\left(t-h\right)$$

Since $K_{0}\left(t\right)=K\left(t-h\right)$, and the Bump function K(·) is non-zero only within the interval (h,2h), during t∈[h,2h], $K_{0}\left(t\right)=0$, and the equation degenerates into an un-delayed homogeneous differential equation ${\dot{e}}_{1,i}\left(t\right)=ae_{1,i}\left(t\right)$. Solving it yields its analytical motion trajectory:

(32) $$e_{1,i}\left(t\right)=e^{a(t-h)}e_{1,i}\left(h\right),\;\forall t\in \left[h,2h\right]$$

This indicates that in this phase, the system error exhibits natural exponential decay, providing a continuous historical state mapping for the delay term in the next phase, derived as $e_{1,i}\left(t-h\right)=e^{a(t-2h)}e_{1,i}\left(h\right)$.

#### 4.2.4. Phase 4: Predefined-Time Forced Convergence Phase (t∈[2h,3h])

During t∈[2h,3h], the artificial delay gain $K_{0}\left(t\right)$ is fully activated. Substituting the derived analytical solution of the delayed state from the previous phase into the error dynamics equation yields

(33) $${\dot{e}}_{1,i}\left(t\right)-ae_{1,i}\left(t\right)=-K_{0}\left(t\right)e^{a(t-2h)}e_{1,i}\left(h\right)$$

Multiplying both sides by the integrating factor $e^{-at}$, it can be transformed into a standard exact differential form:

(34) $$\frac{d}{dt}\left[e_{1,i}\left(t\right)e^{-at}\right]=-e^{-at}K\left(t-h\right)e^{a(t-2h)}e_{1,i}\left(h\right)$$

Substitute the gain function $K\left(t-h\right)=R_{h}\left(t-h\right)e^{ah}We^{-2a(t-h)}$ into the right side of the above equation. The exponential terms can be simplified to $-WR_{h}\left(t-h\right)e^{-2a(t-h)}e^{-ah}e_{1,i}\left(h\right)$. Performing definite integration on this expression over the interval [2h,t] yields

(35) $$e_{1,i}\left(t\right)e^{-at}-e_{1,i}\left(2h\right)e^{-2ah}=-We^{-ah}e_{1,i}\left(h\right)\int_{2h}^{t}R_{h}\left(\tau -h\right)e^{-2a(\tau -h)}d\tau$$

Substituting $e_{1,i}\left(2h\right)=e^{ah}e_{1,i}\left(h\right)$ and multiplying both sides by $e^{at}$, we obtain the exact analytical solution of this delay differential equation during [2h,3h]:

(36) $$e_{1,i}\left(t\right)=e^{a(t-h)}\left[1-W\int_{2h}^{t}R_{h}\left(\tau -h\right)e^{-2a(\tau -h)}d\tau \right]e_{1,i}\left(h\right)$$

Let the integral variable substitution be θ=τ−h. As time approaches the user-predefined boundary t→3h, the upper limit of the integral evolves to 2h:

(37) $$e_{1,i}\left(3h\right)=e^{2ah}\left[1-W\int_{h}^{2h}R_{h}\left(\theta \right)e^{-2a\theta }d\theta \right]e_{1,i}\left(h\right)$$

According to the prior definition of the normalized integral weight $W={\left(\int_{h}^{2h}R_{h}\left(\theta \right)e^{-2a\theta }d\theta \right)}^{-1}$, the time-varying gain term in the square brackets precisely converges to $1-W\cdot W^{-1}=0$. This mathematically guarantees that the tracking error is forcibly truncated at the predefined time t=3h:

(38) $$e_{1,i}\left(3h\right)\equiv 0$$

Thereafter (t≥3h), since the gain $K_{0}\left(t\right)$ returns to zero again, the system maintains ${\dot{e}}_{1,i}=ae_{1,i}$ with the initial value $e_{1,i}\left(3h\right)=0$; thus the origin becomes a stable equilibrium domain.

In conclusion, by combining the projection operator analysis with the deconstruction of nonlinear delay differential equations, it is not only proven that all closed-loop signals of the 2-DOF robotic manipulator are semi-globally uniformly ultimately bounded under parameter perturbations and complex external disturbances, but it is also strictly proven analytically that its error dynamics can perfectly converge to zero at the exact user-predefined time $T_{c}=3h$.

## 5. Simulation and Results

**Figure 2:**
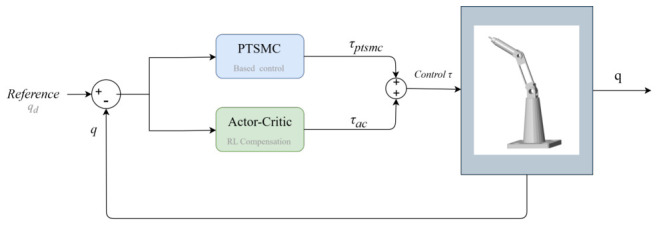
Flowchart of reinforcement learning-based predefined-time sliding mode control.

To concretely verify the effectiveness and performance of the proposed “predefined-time controller integrating reinforcement learning” in computer simulations, a series of detailed numerical simulations are conducted in this section. We select a 2-DOF robotic manipulator model as the simulation object, in which the M(q), $C(q,\dot{q})$, G(q) structure adopted is the standard two-link rigid planar manipulator model introduced in [41] and subsequently adopted by recent learning-based manipulator-control studies such as [16]. The overall control flowchart is shown in Figure 2, and the related dynamic system parameters are set as follows:

(39) $$M\left(q\right)=\left[\begin{array}{cc} p_{1}+p_{2}+2p_{3}\cos q_{2} & p_{2}+p_{3}\cos q_{2}\\ p_{2}+p_{3}\cos q_{2} & p_{2} \end{array}\right]$$

(40) $$C\left(q,\dot{q}\right)=\left[\begin{array}{cc} -p_{3}{\dot{q}}_{2}\sin q_{2} & -p_{3}\left({\dot{q}}_{1}+{\dot{q}}_{2}\right)\sin q_{2}\\ p_{3}{\dot{q}}_{1}\sin q_{2} & 0 \end{array}\right]$$

(41) $$G\left(q\right)=\left[\begin{array}{c} p_{4}g\cos q_{1}+p_{5}g\cos\left(q_{1}+q_{2}\right)\\ p_{5}g\cos\left(q_{1}+q_{2}\right) \end{array}\right]$$

where $p_{1}=m_{1}l_{c1}^{2}+m_{2}l_{1}^{2}+I_{1}$, $p_{2}=m_{2}l_{c2}^{2}+I_{2}$, $p_{3}=m_{2}l_{1}l_{c2}$, $p_{4}=m_{1}l_{c1}+m_{2}l_{1}$, and $p_{5}=m_{2}l_{c2}$. The physical parameters are defined as follows: $m_{i}$ and $l_{i}$ denote the mass and length of link *i*, respectively, with $m_{1}=m_{2}=1.0$ kg and $l_{1}=l_{2}=1.0$ m; g=9.81 m/s^2^ is the gravitational acceleration; $I_{i}$ is the moment of inertia of link *i*, calculated as $I_{1}=\frac{1}{4}m_{1}l_{1}^{2}$ kg·m^2^ and $I_{2}=\frac{1}{4}m_{2}l_{2}^{2}$ kg·m^2^; and $l_{ci}$ represents the distance from joint i−1 to the center of mass of link *i*.

To verify the robustness of the proposed PTC-RLC strategy under significant parameter perturbations, the nominal mass used by the controller is intentionally set as $m_{10}=m_{20}=1.8$ kg, representing an 80% deviation from the actual link mass $m_{1}=m_{2}=1.0$ kg. Furthermore, the lumped disturbance d(t) in Equation (1) is modeled as a combination of viscous friction, Coulomb friction, and time-varying external disturbances:

(42) $$d\left(t\right)=0.5\dot{q}+0.5\mathrm{sgn}\left(\dot{q}\right)+\left[\begin{array}{c} 2.0\sin(t)\\ 2.0\cos(t) \end{array}\right]$$

The simulation is conducted with a sampling period of dt=0.001 s. To ensure reproducibility and enable a clear comparison, the specific control gains, RBF neural network hyperparameters, and predefined time constant are summarized in Table 1. These parameters are selected to balance convergence speed and control smoothness.

The parameters of the NTSMC are tuned to achieve its best possible performance under the same disturbance conditions to ensure a fair comparison.

### 5.1. Trajectory Tracking Performance and Control Input Analysis

To comprehensively evaluate the overall performance of the proposed predefined-time sliding mode controller integrating reinforcement learning (PTC-RLC, denoted as Proposed (RL) in the figures), this section presents a detailed comparative analysis with a traditional baseline controller (Baseline) and a non-singular terminal sliding mode controller (NTSMC) [42]. In the comparative simulations, the “Baseline” controller is specifically defined as the proposed predefined-time sliding mode control architecture without the auxiliary Actor–Critic reinforcement learning compensation network (i.e., by enforcing $f_{NN}\equiv 0$). This ablation setup isolates and clarifies the unique contribution of the reinforcement learning agent in dynamically mitigating unknown parameter variations and external disturbances. Regarding the choice of external comparison, NTSMC [42] is selected as it represents a state-of-the-art finite-time robust controller widely benchmarked in the manipulator-control literature. A classical PID controller was preliminarily evaluated under the same operating conditions but is omitted from the comparative figures, because under the imposed 80% mass mismatch and the time-varying disturbance d(t) specified below, the PID controller exhibits sustained tracking errors and visible drift, which renders it uncompetitive against any sliding-mode-based scheme and offers limited additional insight into the chattering-suppression and predefined-time properties that are the central focus of this paper.

**Figure 3:**
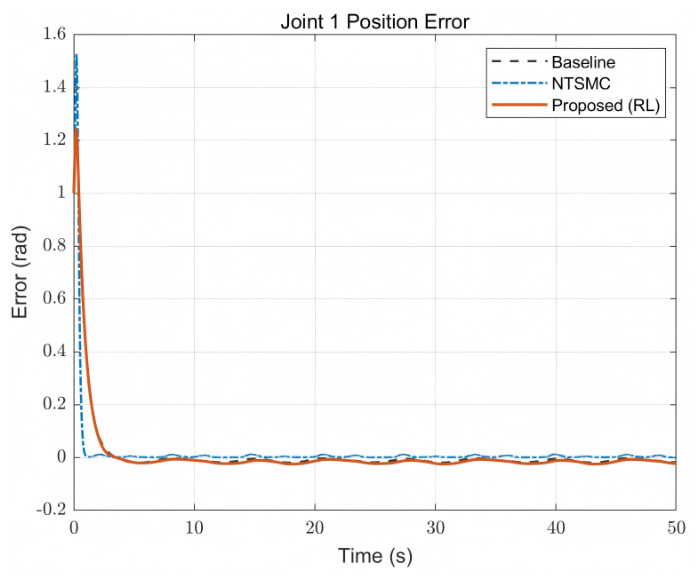
Angle tracking error comparison for Joint 1.

**Figure 4:**
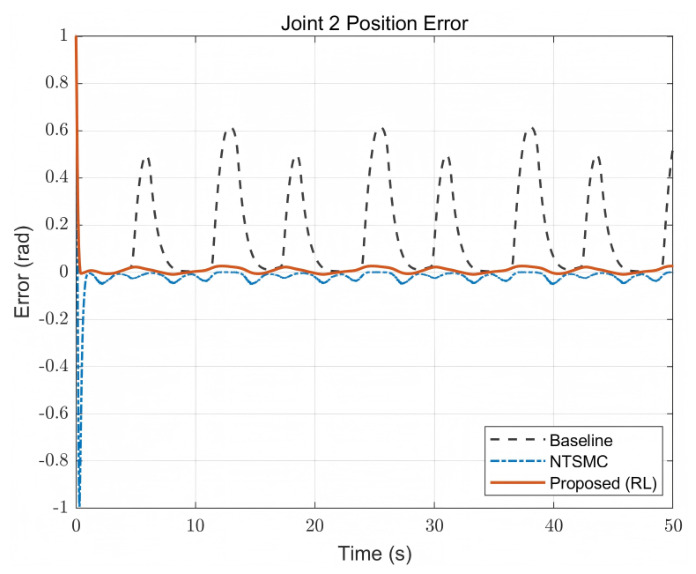
Angle tracking error comparison for Joint 2.

Figure 3 and Figure 4 show the trajectory tracking curves of Joint 1 and Joint 2 of the robotic manipulator, respectively. Under the harsh operating conditions of strong parameter perturbations and external disturbances, all three controllers track the desired trajectory. However, the Baseline controller exhibits noticeable phase lag during the initial response phase and reduced fidelity where curvature changes rapidly, such as at the trajectory peaks and valleys. NTSMC improves the response speed to a certain extent, but visible trajectory deviations remain. In contrast, the proposed scheme converges to the reference trajectory faster, exhibits the predefined-time convergence behavior, and maintains close trajectory overlap throughout the motion cycle with no apparent lag or overshoot.

**Figure 5:**
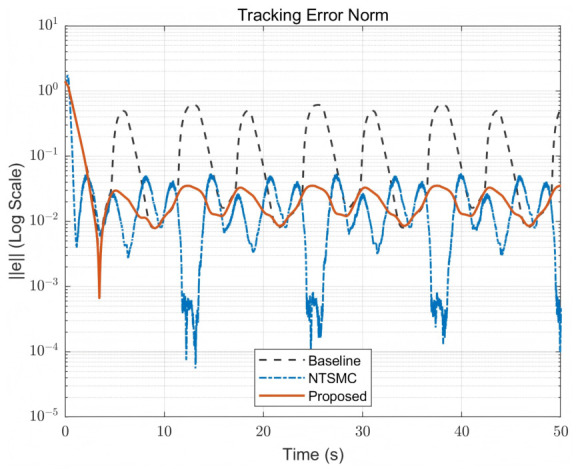
Comparison of the system total tracking error norm under three controllers.

To intuitively evaluate the overall control precision of the system in both transient and steady states, Figure 5 displays the evolution curve of the comprehensive position tracking error norm ∥e∥ for the dual joints. To clearly reveal the fluctuation details of microscopic errors, this curve is plotted using a log scale.

As shown in Figure 5, in the initial transient phase, the error of the Baseline controller converges the slowest, and although NTSMC converges faster, its decay rate slows down noticeably as it approaches zero. The proposed method (Proposed) exhibits the fastest error decay, and within the prescribed time $T_{c}=4.5$ s, the error converges to a neighborhood of zero, confirming that the predefined-time sliding surface delivers convergence within the designed time window. After entering the steady-state tracking phase, due to the model parameter uncertainties and external disturbances, the error of the Baseline controller fluctuates noticeably and remains at the order of $10^{-1}$. NTSMC reduces the overall error, but its curve shows high-frequency spikes, reflecting the chattering caused by the high-gain switching term. The proposed method, by employing the Actor–Critic reinforcement learning network for online estimation and compensation of disturbances, reduces the controller’s reliance on large switching gains; consequently, the steady-state error is kept below the order of $10^{-2}$ and the curve is smooth, substantially suppressing the chattering and yielding improved control precision.

**Figure 6:**
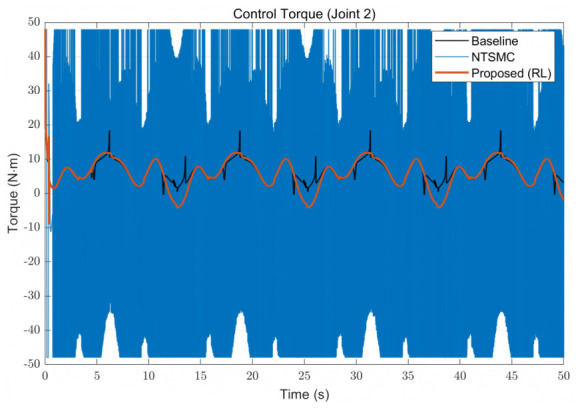
Comparison of control input torques for the robotic manipulator joint under three different controllers.

The input torque comparison curves of the three controllers are shown in Figure 6. NTSMC, in resisting strong disturbances through high-gain switching, produces control torques accompanied by visible high-frequency chattering. Such high-frequency oscillations can excite unmodeled high-frequency dynamics of the manipulator in actual physical systems and cause wear on actuators such as motors. The proposed strategy outputs a smoother, continuous torque curve that mitigates the inherent chattering of traditional sliding mode control; the torque amplitude is kept within a reasonable physical range with no abrupt jumps, while the high tracking precision reported above is preserved. This indicates that the algorithm has good engineering implementation feasibility while delivering the transient and steady-state performance reported above.

Furthermore, to evaluate the real-time industrial applicability, the computational cost of the proposed control law was quantified. All timing experiments were conducted on a desktop workstation equipped with an Intel Core i5-14600KF processor running Windows 10 and MATLAB R2024a. The average single-step execution time was measured using MATLAB’s tic/toc utilities, with the timer wrapping only the controller-update block of each iteration and the recorded times averaged over the entire 50,000-step simulation horizon ($T_{\mathrm{final}}=50$ s with sampling period $dt=10^{-3}$ s). Under this protocol, the proposed PTC-RLC algorithm requires approximately 0.0143 ms per control cycle, which is nearly two orders of magnitude below the 1 ms budget of a standard 1 kHz industrial control loop. Because the measurement is obtained inside an interpreted MATLAB environment, an equivalent C/C++ implementation deployed on industrial embedded hardware would be expected to execute substantially faster. This minimal computational overhead indicates that the proposed algorithm is suitable for real-time deployment at standard industrial control frequencies without inducing control delays.

### 5.2. Performance Evaluation Under Extreme Trajectories

To further thoroughly evaluate the performance limits, transient overshoot, and switching behavior of the proposed PTC-RLC strategy, two additional extreme trajectory cases are conducted: a high-frequency continuous trajectory and a point-to-point step response. The high-frequency trajectory is utilized to test the bandwidth and convergence limits of the predefined-time sliding surface, while the step response, characterized by a massive initial error, is implemented to observe the overshoot and dynamic reaching process of the controller. It should be noted that this subsection focuses on characterizing the intrinsic bandwidth, overshoot, and switching behavior of the proposed PTC-RLC under extreme dynamic conditions, rather than repeating the three-controller comparison of Section 5.1. Comparative curves with NTSMC under the step input are not reported because the discontinuous high-gain switching of NTSMC induces severe transient chattering and torque saturation at the instant of the step jump, leading to non-representative actuator behavior; under the high-frequency reference, NTSMC was preliminarily verified to be inferior to the proposed scheme with steady-state error exceeding $5\times 10^{-2}$ rad, and is omitted to preserve figure clarity.

**Figure 7:**
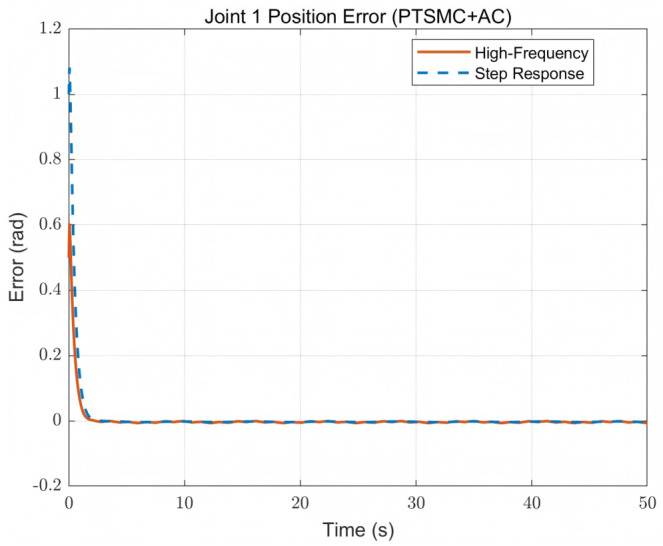
Position error comparison of Joint 1 under high-frequency and step response trajectories.

**Figure 8:**
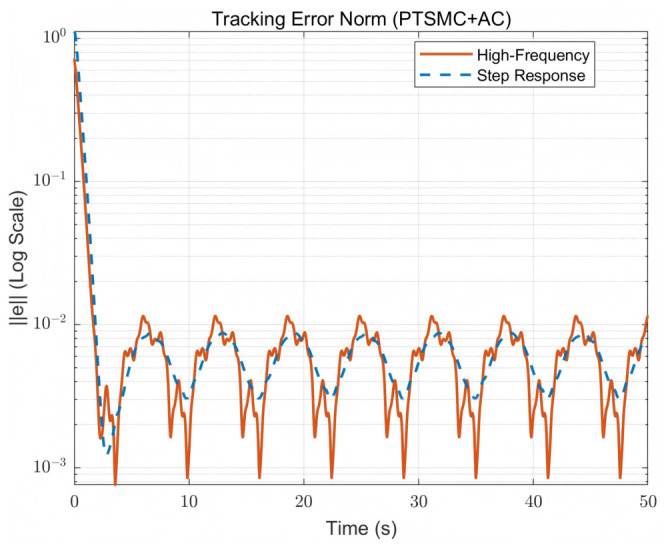
System tracking error norm (log scale) under different trajectory conditions.

As depicted in Figure 7 and Figure 8, in the point-to-point step response scenario (dashed blue line), the proposed controller demonstrates an exceptionally smooth transient reaching behavior. Despite the sudden and large initial position deviation, the error strictly converges to zero without any noticeable overshoot. This fully validates that the constructed sliding surface based on smooth artificial delay feedback effectively suppresses the excessive transient chattering commonly observed in conventional reaching laws. In the high-frequency trajectory tracking scenario (solid red line), the controller exhibits strong tracking capability. As shown in the logarithmic error norm in Figure 8, the steady-state tracking error is firmly confined to a minimal scale (<$10^{-2}$).

**Figure 9:**
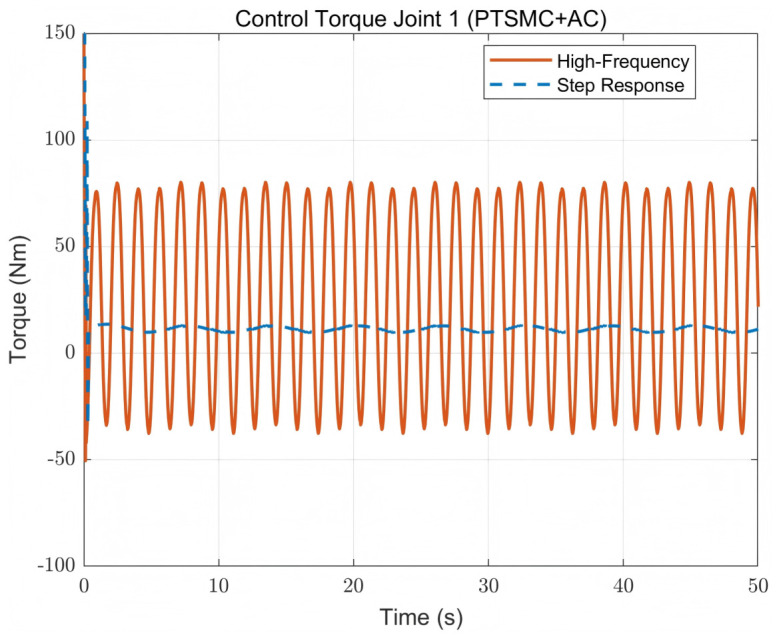
Control torque input of Joint 1 under extreme trajectory conditions.

Furthermore, Figure 9 illustrates the control torque behavior under these extreme conditions. For the step response, the torque naturally peaks at the initial moment to drive the manipulator toward the target but rapidly decays to a smooth, steady-state level compensating only for gravity and friction. In contrast, for the high-frequency trajectory, the controller must output high-frequency oscillating torque to force the mechanical structure to track rapidly changing references. Even under such aggressive dynamic demands, the torque remains bounded and does not exhibit high-frequency infinite chattering, confirming the controller’s reliability under extreme dynamic limits.

### 5.3. Effect of the Predefined-Time Parameter $T_{c}$

One of the most significant theoretical advantages of the proposed method is that the convergence time can be explicitly scheduled by adjusting the single parameter $T_{c}$. To visually demonstrate how the controller behaves with different predefined-time parameters, an additional comparative study is performed using the step response trajectory, evaluating the convergence behavior under $T_{c}\in \left\{1.0\;s,1.5\;s,2.0\;s\right\}$.

**Figure 10:**
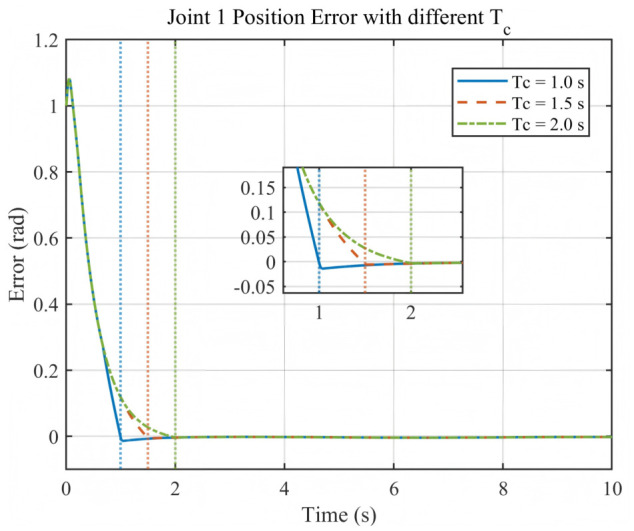
Position tracking error of Joint 1 under varying predefined-time parameters $T_{c}$. The vertical dotted lines represent the explicitly set convergence deadlines.

The simulation results are comprehensively presented in Figure 10. The vertical dotted lines with corresponding colors mark the exact prescribed deadlines $T_{c}$. It is evident that regardless of the chosen $T_{c}$, the tracking error curves successfully hit the equilibrium point (zero) exactly at or slightly before their respective predefined times. Specifically, when $T_{c}=2.0\;s$, the error decays smoothly; when a more aggressive parameter $T_{c}=1.0\;s$ is selected, the convergence rate dramatically accelerates. A slight numerical residual with peak magnitude below 0.05 rad is observed at $t\approx T_{c}$ for the most aggressive setting. This residual is attributable to the discrete-time numerical implementation with the finite sampling period $dt=10^{-3}$ s, which limits the achievable resolution of the smooth Bump kernel $R_{h}\left(t\right)$ when its compact support (h,2h) shrinks to a narrow time window of approximately 0.33 s and is thereby covered by only a few hundred sampling instants; the residual decreases monotonically as dt→0, consistent with the analytical convergence guarantee in Theorem 1. The error is immediately trapped in the sliding manifold thereafter. This explicit parameter-adjustment mechanism provides great flexibility for practical engineering deployments, allowing operators to make a reasonable trade-off between strict convergence time and acceptable actuator torque limits.

## 6. Conclusions

Targeting the trajectory tracking problem of rigid robotic manipulators affected by model uncertainties and time-varying complex disturbances, this paper proposes a composite control strategy integrating Actor–Critic reinforcement learning and predefined-time sliding mode (PTC-RLC). By constructing a predefined-time sliding surface based on artificial delay feedback, the proposed method avoids the singularity issue caused by fractional-order terms in traditional fixed-time control, and a Lyapunov-based analysis shows that the tracking error converges within a user-prescribed time $T_{c}$ independent of the initial states. The proposed controller architecture generalizes naturally from the 2-DOF illustrative case to *n*-DOF manipulators: since the sliding-surface design and the Actor compensator are constructed on a per-joint basis, both the per-joint sliding-surface computation and the Actor network scale linearly with the number of joints, avoiding the exponential growth in computational load that typically accompanies the addition of degrees of freedom; for high-DOF systems, a corresponding decomposition of the Critic network can preserve this linear scaling. The Actor–Critic network in the control loop provides online approximation and dynamic compensation of lumped unknown dynamics, which reduces the controller’s reliance on high robust switching gains. Theoretical analysis establishes the semi-global uniform ultimate boundedness of all signals within the closed-loop system. Numerical simulation results show that under parameter mismatch and time-varying disturbances, the proposed PTC-RLC strategy maintains predefined-time trajectory tracking. Compared to a representative non-singular terminal sliding mode controller (NTSMC), the proposed strategy suppresses the high-frequency chattering of the control input by leveraging the dynamic compensation provided by reinforcement learning. The resulting smoother control torque is expected to reduce mechanical wear of actuators in physical systems, suggesting that the method has potential for future practical deployment; this expectation remains to be confirmed through physical experimental verification.

## Figures and Tables

**Table 1:** Parameters of the controllers and RL networks.

Category	Parameter	Value
Predefined-time Control	Predefined time $T_{c}$	4.5 s
	Sliding surface gain *a*	−1.5
	Robust switching gain $k_{rob}$	9.9
	Exponent parameters λ,μ	3, 1.2
	Boundary layer ϵ	0.05
Actor–Critic RL	Actor learning rate $\eta_{a}$	10.0
	Critic learning rate $\eta_{c}$	30.0
	Weight constraint $\bar{W}$	50.0
	RBF hidden nodes *N*	25
	Gaussian basis width ω	0.4
NTSMC (Baseline)	Gain parameters α,β,γ	10, 1/3, 5/3
	Reaching gains $k_{1},k_{2}$	30, 10

## Data Availability

The data presented in this study are available on request from the corresponding author.
